# Insights into child abuse and neglect: Findings from the Minnesota Longitudinal Study of Risk and Adaptation

**DOI:** 10.1017/S0954579424000865

**Published:** 2024-04-22

**Authors:** Marissa D. Nivison, Madelyn H. Labella, K. Lee Raby, Jenalee R. Doom, Jodi Martin, William F. Johnson, Osnat Zamir, Michelle M. Englund, Jeffry A. Simpson, Elizabeth A. Carlson, Glenn I. Roisman

**Affiliations:** 1 Institute of Child Development, University of Minnesota Twin Cities, Minneapolis, MN, USA,; 2 Psychological Sciences, William & Mary, Williamsburg, VA, USA; 3 Department of Psychology, University of Utah, Salt Lake City, UT, USA; 4 Department of Psychology, University of Denver, Denver, CO, USA; 5 York University, Toronto, ON, USA; 6 Old Dominion University, NorfolkNFK, VA, USA; 7 Social Work, Hebrew University, Jerusalem, SR, USA; 8 Department of Psychology, University of Minnesota Twin Cities, Minneapolis, MN, USA

**Keywords:** Child abuse and neglect, maltreament, prospective longitudinal study

## Abstract

The Minnesota Longitudinal Study of Risk and Adaptation (MLSRA) is a landmark prospective, longitudinal study of human development focused on a sample of mothers experiencing poverty and their firstborn children. Although the MLSRA pioneered a number of important topics in the area of social and emotional development, it began with the more specific goal of examining the antecedents of child maltreatment. From that foundation and for more than 40 years, the study has produced a significant body of research on the origins, sequelae, and measurement of childhood abuse and neglect. The principal objectives of this report are to document the early history of the MLSRA and its contributions to the study of child maltreatment and to review and summarize results from the recently updated childhood abuse and neglect coding of the cohort, with particular emphasis on findings related to adult adjustment. While doing so, we highlight key themes and contributions from Dr Dante Cicchetti’s body of research and developmental psychopathology perspective to the MLSRA, a project launched during his tenure as a graduate student at the University of Minnesota.

The Minnesota Longitudinal Study of Risk and Adaptation (MLSRA) is a prospective, longitudinal investigation of human development that has followed at-risk mothers and their first-born children from three months before birth through approximately 40 years of age (with data collection currently ongoing in the adult children’s mid to late 40s). The MLSRA was and remains a pioneering study in the field of developmental psychology and is regarded as one of the most important prospective longitudinal studies of human development to date (Duschinsky, 2020). Although initially designed to examine the etiology of childhood maltreatment, the MLSRA has contributed to the literature on developmental psychology literature in a number of areas, including infant–mother attachments (e.g., Egeland & Sroufe, 1981; Egeland & Farber, 1984), peer and romantic relationship functioning (e.g., Collins et. al., 1997), physical and mental health (e.g., Puig et al., 2013; Sroufe, 1997), and academic and work performance (Collins & van Dulmen, 2006; Raby et al., 2014) through midlife. The goals of this narrative review are to return to the maltreatment origins of the project to summarize the early history of the MLSRA and some of its contributions to the field of child maltreatment and to synthesize key findings based on recently updated coding of childhood abuse and neglect experienced by some of the now adult children who have participated in this study for almost five decades. Throughout this review, we highlight key themes and significant contributions from the maltreatment research and the developmental psychopathology perspective of Dr Dante Cicchetti to the MLSRA.

## A history of the Minnesota Longitudinal Study of Risk and Adaptation (MLSRA) and its contributions to the maltreatment literature

The MLSRA was conceived and initially conducted by Dr Byron Egeland in collaboration with a pediatrician, Dr Amos Deinard, to better understand the origins of caregiver–child maltreatment (Sroufe et al., 2005). While the cohort was still in childhood, Byron Egeland was joined by Drs. L. Alan Sroufe, Andrew Collins, Elizabeth Carlson, and Michelle Englund and, later still (when the target participants reached adulthood), by Dr Jeff Simpson and Dr Glenn Roisman. Drawing on risk designs originating in schizophrenia research (e.g., Garmezy, 1975), the MLSRA enrolled participants who were living at or below the poverty line at the time of recruitment. Although it was not known whether the child participants in the sample would go on experience maltreatment, Drs. Egeland and Deinard expected that, by recruiting from an at-risk population, the rates of maltreatment in this sample would be higher than that observed in the general population (Sroufe et al., 2005). From the early days of the project and over the subsequent 40+ years, the study has contributed to the maltreatment literature by (1) examining the antecedents of maltreatment (and challenging existing assumptions in the literature), (2) advancing research on the sequelae of maltreatment, and (3) exploring the intergenerational transmission of maltreatment. Major findings across these domains are reviewed below.

## Examining the antecedents of maltreatment and challenging assumptions in the literature

The early findings of the MLSRA challenged several existing assumptions about maltreatment in the field at the time (e.g., Belsky, 1993). In the early 1970s, common assumptions included the idea that maltreatment was caused by the stresses of poverty, child characteristics (e.g., temperament, preterm birth), and parental personality traits (Egeland, 1979). However, the early literature on child maltreatment relied heavily on retrospective, cross-sectional studies that did not allow for the precise study of childhood maltreatment antecedents, and these assumptions had not been rigorously tested through longitudinal research (Egeland, 1979). In contrast, the MLSRA prospective longitudinal study starting prior to childbirth allowed examining multiple potential etiological factors versus a single casual factor that was likely to fail to fully explain why children experienced maltreatment (Egeland, 1979, p. 270). Based on these ideas, the MLSRA recruited 267 primiparous mothers who were receiving prenatal care from public health clinics in Minneapolis, Minnesota. The MLSRA team focused on three main factors as potential antecedents of childhood maltreatment: environmental and sociological stress, parent–child interactions, and child traits or characteristics.

The MLSRA team conducted multiple waves of assessment, beginning with interviews with the mothers late in their pregnancies (i.e., approximately 36 weeks), nurses’ ratings of the infants at birth, and extensive home visits during the child’s first year of life. During these early years, childhood maltreatment was assessed through multiple measures that included parent interviews and home observations based on the Child Care Rating Scale (Egeland & Deinard, 1975). The Child Care Rating Scale included indicators related to evidence of violence in the household (in particular, violence directed toward the child), poor physical care of the child, inadequate living conditions, and child neglect or failure to thrive. Four principal patterns of maltreatment were identified from these assessments: physical abuse, physical neglect, psychological unavailability (i.e., emotional neglect), and verbal abuse; patterns of sexual abuse were included later in the study (Sroufe et al., 2005). The overall maltreatment rate in the MLSRA sample from assessments over the first 24 months of the children’s lives was approximately 10%, a rate significantly higher than that reported for the city of Minneapolis at the time (1%–2%; Egeland & Brunnquell, 1979).

Early results from the MLRSA revealed that maltreatment of these young children was more likely to occur among younger mothers, who were less educated, and/or less knowledgeable about the psychological complexity of their infant (i.e., mothers who either viewed their infants in a wholly positive or negative light), and who received less social support (Sroufe et al., 2005). There was little evidence to suggest that mothers’ personality traits (e.g., anxiety, aggression, impulsivity) or an infant’s preterm birth were associated with later maltreatment, contrary to assumptions in the field at the time. Furthermore, there was little evidence to suggest that a child’s traits (e.g., temperament) elicited maltreatment. Instead, the MLSRA investigators found that the overall quality of mother–child interaction during feeding and free play at ages 3 and 6 months was a significant predictor of later maltreatment in that mother–infant pairs that displayed higher quality interactions (e.g., positive regard, low hostility) were less likely to maltreat their infants. Egeland and Brunnquell (1979) recommended that observing mother–infant interactions would be a much more valid assessment of maltreatment risk than the “paper-and-pencil screening devices” which were prevalent at the time (Egeland & Brunnquell, 1979). These findings were pivotal in shifting the way in which the etiology of childhood maltreatment was viewed within the field.

Given these early MLSRA maltreatment findings, when the children turned 18 months of age, plans were developed to follow the sample into early childhood to better understand the effects of individual differences in caregiving on child development more broadly. As a result, the MLSRA moved to examining maltreatment as a predictor variable, instead of only an outcome.

## The sequelae of maltreatment and the transition into a much broader study

Although considerable research had focused on the consequences of maltreatment, up to the early 1970s, only a few studies utilized prospective longitudinal data from an at-risk sample. Therefore, the MLSRA team adopted a developmental psychopathology approach (Sroufe & Rutter, 1984; Cicchetti, 1993) to studying development. This perspective incorporates both normative and atypical life experiences, multiple levels of analysis (e.g., psychosocial, biological), and a range of assessment strategies (e.g., parent-report, teacher-report, direct observation). Guided by fellow developmental psychopathologists, this approach led to more rigorous investigations of maltreatment as well as investigating a wider range of developmental phenomena through the MLSRA and related studies (see Cicchetti, 2004). The way in which the MLSRA investigated the sequelae of maltreatment was based on the existing work of fellow developmental psychopathologists, primarily Dr Dante Cicchetti, as noted by Byron Egeland et al. (2002; p. 250):

“We, along with other investigators, have been guided in our study of the sequelae of maltreatment by an organizational perspective of development that focuses on the qualitative reorganization and integration that takes place within and among behavioral and biological systems of the individual (Cicchetti, 1993; Sroufe & Rutter, 1984).”

Studies of early sequelae of maltreatment in the MLSRA included an examination of the effect of childhood maltreatment on infant attachment quality (Carlson, 1998; Egeland & Sroufe, 1981; see also Carlson et al., 1989). The finding that infants who had experienced any type of maltreatment during the first year of life were at increased risk for attachment relationship disturbance (i.e., disorganization) at 12 and 18 months of age advanced an understanding of the impact of early maltreatment and the formation and role of early caregiving relationships in development. The MLSRA staff continued to investigate the sequelae of maltreatment on well-being across childhood and adolescence. In toddlerhood, for example, children with histories of any form of maltreatment showed lower levels of persistence and enthusiasm as well as higher levels of inattention and negative affect during mildly challenging tasks during the preschool period (Egeland et al., 1983). Preschool-aged children with histories of maltreatment had lower self-esteem and agency during a problem-solving task (i.e., Barrier Box assessment) when compared with non-maltreated children (Egeland et al., 1983). Upon starting school, children with maltreatment histories were disadvantaged, demonstrating poorer language skills, having greater difficulty following directions, struggling to work independently, and not persisting on challenging tasks (Egeland, 1988; Erickson et al., 1989). During childhood, maltreatment experiences were also linked to higher rates of behavior problems, especially for boys (Erickson et al., 1989), and greater depressive symptomatology (Duggal et al., 2001). In adolescence, children with maltreatment histories were more likely to receive a psychiatric diagnosis (Egeland, 1997) and engage in self-injurious behavior (Yates, 2004).

Consistent with recommendations in the maltreatment literature (Barnett et al., 1993) and in view of all the data that had been collected, the MLSRA next examined the sequelae of specific subtypes of maltreatment across childhood and adolescence. For example, during the preschool period, children with histories of physical abuse were more likely to be distracted and experience negative affect, whereas those with neglect histories were more likely to withdraw from social experiences (Egeland et al., 1983). Further, a history of physical abuse was associated with being diagnosed with conduct disorder and oppositional defiant disorder; a history of neglect or sexual abuse increased the likelihood of an anxiety disorder diagnosis; and a history of emotional unavailability or sexual abuse was associated with increased risk of posttraumatic stress disorder (Egeland, 1997). Rates of comorbid disorders (i.e., two or more simultaneous psychiatric disorders) by maltreatment type in MLSRA were as follows: 73% of sexual abuse cases, 54% of neglect cases, 60% of physical abuse cases, and 73% of psychological unavailability cases (groups not mutually exclusive). In contrast, 30% of participants with no history of maltreatment met the criteria for multiple psychiatric disorders, a rate consistent with general poverty risk estimates at that time (Egeland, 1997).

## The intergenerational transmission of maltreatment

When the MLSRA children were 48–54 months of age, retrospective interviews were conducted with their mothers about their own childhood experiences of maltreatment (Egeland et al., 1988). The interviews focused on the degree of caregiver support received as a child, how affection was expressed, forms of discipline used within the family, and whether they were physically or sexually abused or neglected. Based on interview responses, overall maltreatment rates and intergenerational concordance of maltreatment were examined. In this study, mothers reporting maltreatment history were more likely to maltreat their children than mothers who did not report experiencing childhood maltreatment (Egeland et al., 1988). Specifically, 40% of mothers with maltreatment histories were found to maltreat their children, and 30% provided borderline care (i.e., suspected maltreatment). There were also lawful changes in the intergenerational patterns of maltreatment. Mothers who broke the intergenerational transmission of maltreatment (i.e., mothers with histories of maltreatment who did not mistreat their children) were more likely to have received emotional support from a non-abusive adult during childhood, to have participated in therapy for at least six months at some point in their lives, or to have an emotionally supportive and satisfying relationship in adulthood (Egeland et al., 1988; see also Egeland & Susman-Stillman, 1996). Mothers who continued the cycle of maltreatment experienced greater life stress and were more likely to be anxious, dependent, immature, or depressed (Egeland et al., 1988). The findings have contributed to prevention and intervention efforts to mitigate the effects of childhood maltreatment across the life span.

In sum, early research from the MLSRA contributed to ways in which scholars, clinicians, and policymakers understood the origins of childhood maltreatment. Additionally, the MLSRA has been pivotal in advancing a more complex understanding of factors that may disrupt the intergenerational transmission of maltreatment experiences and promote responsive care and the effects of maltreatment on individuals’ functioning during infancy, childhood, and adolescence. Grounded in a developmental psychopathology perspective (Cicchetti & Cohen, 1995; Sroufe & Rutter, 1984), the MLSRA has been and continues to be a productive and informative landmark longitudinal study.

## The influence of developmental psychopathology and Dr. Dante Cicchetti on the MLSRA

Throughout the longitudinal research project, the MLSRA has been influenced by the key principles of developmental psychopathology—specifically Dr Cicchetti’s work on understanding both normative and atypical development and the interplay between the two (Cicchetti & Cohen, 1995). This perspective inspired the MLSRA to collect data at multiple levels of analysis (e.g., physical health, psychosocial factors) through multiple methods (e.g., mother- and self-report, home and laboratory observations) across the life span. Furthermore, the MLSRA also adopted methodological designs established by Dr Cicchetti in the Rochester studies at Mt. Hope (i.e., summer camps; Cicchetti et al., 1990). The summer camp design provided a more ecologically valid assessment of children outside of the home and laboratory; it also provided a fun experience for the children and daycare for the parents. By having a subset of the participants in one location, the research team could also assess more relational measures (e.g., social competence and peer relations). More recently, Dr Cicchetti was critical in supporting the DNA collection at the age 32-year assessment and the examination of gene by environment interactions in the MLSRA (e.g., Cutuli et al., 2013).

The guiding principles of the developmental psychopathology perspective have continued to shape the design of the MLSRA assessments through the 40+ years of the project. In 2013, however, Dr Cicchetti’s work on the measurement of childhood maltreatment became pivotal to the project (e.g., Barnett et al., 1993). Despite the extensive prospectively assessed data on childhood maltreatment in the MLSRA, early measurement variability led to challenges in conducting analyses of the cumulative effects of childhood maltreatment on adult functioning. Following several consultations with Dr Cicchetti, the MLSRA maltreatment data were re-coded to apply contemporaneous definitions of abuse and neglect, to identify specific perpetrators, and clarify the timing of abuse and neglect experiences, and to assess the reliability of those re-coding decisions. Drawing on guidelines established by Barnett et al. (1993), MLSRA maltreatment data were reviewed and revised to include clearer definitions and more specific parameters of abuse (e.g., perpetrator, subtype, chronicity). The MLSRA greatly benefited from the groundbreaking work by Dr Cicchetti and colleagues on the clarification of defining childhood maltreatment. We now outline these classification revisions and related research findings.

## Prospective maltreatment (abuse/neglect) recoded data and recent findings

### Newly recoded data

The recoding project to create a more consistent and detailed measure of abuse/neglect experiences across childhood began in 2013 and was completed in 2016 and was led by Drs. Madelyn Labella and Lee Raby (who were graduate students at the time) in consultation with Dr Michelle Englund. As part of this recoding effort, the MLSRA has used the term “childhood experiences of adverse caregiving” as a broad term to refer to a range of atypical parent–child experiences prospectively measured in the MLSRA and believed to be harmful to child development. Information was collected about MLSRA participants’ adverse caregiving experiences of physical abuse, sexual abuse, and neglect. Although emotional unavailability (i.e., lack of caregiver responsiveness) is an important dimension of adverse caregiving that has pernicious developmental consequences, especially for young children (National Scientific Council on the Developing Child, 2012; Sroufe et al., 2005), this variable was not included in the recent coding effort due to insufficient information across developmental periods. Similarly, exposure to violence between caregivers and other forms of environmental violence were not included in the revised set of codes. That said, exposure to violence between caregivers was captured by a separate variable in the MLSRA data set (e.g., Narayan et al., 2013), though insufficient information was available to code exposure to other forms of environmental violence adequately.

The revised coding criteria were, in part, based on definitions developed by the Centers for Disease Control and Prevention (CDC) to “promote consistent terminology and data collection related to child maltreatment” (Leeb et al., 2008, p. 4). The coding included: (1) neglect of a child’s basic physical or cognitive needs, defined as a caregiver’s failure to provide adequate hygiene, shelter, clothing, medical care, supervision, or education, (2) physical abuse, defined as a caregiver’s “intentional use of physical force against a child that results, or has the potential to result in, physical injury” (Leeb et al., 2008, p. 14), and (3) sexual abuse, defined as sexual contact (e.g., molestation, rape) or noncontact exploitation (e.g., intentional exposure of a child to pornography) by a custodial caregiver or a perpetrator five or more years older than the child. Although the CDC criteria address only sexual abuse perpetrated by a caregiver, the inclusion of non-caregiving perpetrators and the use of a 5-year cutoff is consistent with other research in this area (e.g., Stoltenborgh et al., 2011).

The CDC maltreatment definitions were supplemented with a set of more specific coding guidelines that distinguished clear indicators of physical abuse, sexual abuse, and physical/cognitive neglect from ambiguous indicators that were insufficient for classification without other evidence. These additional guidelines were developed in consultation with MLSRA researchers, Minnesota state law, and the available research literature (e.g., Barnett et al., 1993) and are available from the first author upon request. The classifications of childhood experiences of abuse or neglect, however, do not necessarily reflect the criteria for maltreatment used by Child Protective Services (CPS), which vary from state to state. As such, our scoring of abuse and neglect does not necessarily mean that these children or their families were actively involved with CPS.

For the recoding project, all available data collected from birth to 17.5 years (up to 25 assessments) were reviewed for information regarding caregiving quality, physical discipline, supervision, home environment, physical and sexual assault, CPS involvement, and foster care history. Information was obtained from parent–child observations, caregiver interviews, reviews of available child protection and medical records, adolescent reports, and teacher interviews.

Coding focused on the presence or absence of physical abuse, sexual abuse, and/or neglect during each of four developmental periods (Infancy: birth to 24 months; Early Childhood: 25 months to five years; Middle Childhood: 6–12 years; and Adolescence: 13–17.5 years). For incidents of physical and sexual abuse, coders also specified the perpetrator. Perpetrators included maternal caregivers (biological mothers, stepmothers, and grandmothers), paternal or father figures (biological fathers, stepfathers, adoptive fathers, and mothers’ live-in boyfriends), and non-parental figures (relatives, neighbors, babysitters, and family friends). Two coders reviewed each case and demonstrated good-to-excellent reliability for all parameters: kappas ranged between .80 and .98 for presence or absence of physical abuse, sexual abuse, and/or neglect, between .80 and .84 for presence or absence of each type during each development period; and between .80 and .98 for incidents of physical or sexual abuse by each category of perpetrator. All discrepancies were resolved by consensus.

Within the full sample of MLSRA participants (*N* = 267), 102 individuals were classified as having ever experienced physical abuse, sexual abuse, and/or neglect; 81 were coded as not having experienced abuse or neglect; and the status of 84 was deemed unclear due to missing data. By developmental period, individuals identified as maltreated (abused/neglected) included 47 individuals in infancy (of the 211 with sufficient data for confident classification during this developmental period), 66 in early childhood (of the 185 with sufficient data during this developmental period), 66 in middle childhood (of the 190 with sufficient data during this developmental period), and 21 in adolescence (of the 179 with sufficient data during this developmental period). These newly recoded data have provided the basis for a series of recent MLSRA research projects investigating the legacy of childhood abuse/neglect across the life span, which are summarized below.

## Summary of the findings of research using the new maltreatment coding

Based on the 2016 MLSRA maltreatment codes and guidelines, recent publications based on the MLSRA have examined the associations between child abuse/neglect and adult attachment, adult romantic functioning, adult psychopathology, second-generation parenting, life span academic and social outcomes, and adult physical health. Study descriptions, including the types of abuse/neglect variables and outcomes examined in each study, are presented in Table 1.

### Child abuse/neglect and adult attachment

Raby et al. (2017) examined the extent to which experiences of childhood abuse and/or neglect were associated with later adult attachment representations, measured both in the context of global attachment representations (via the Adult Attachment Interview [AAI]; Main et al., 1985) and in the context of romantic relationships (via the Current Relationships Interview [CRI]; Crowell & Owens, 1998). The primary analyses operationalized child abuse and/or neglect as an overall binary variable of whether any kind of abuse/neglect had occurred at any time, by any perpetrator from birth to 17.5 years. Following recommendations from the maltreatment literature (e.g., Cicchetti, 2016), Raby et al. (2017) also examined specific parameters of abuse/neglect including: the chronicity of abuse/neglect, the total number of subtypes of abuse/neglect, the subtype of abuse/neglect, and the perpetrator of the abuse. *Chronicity of abuse/neglect* was defined as the number of developmental periods from 0–4 (i.e., infancy, early childhood, middle childhood, adolescence) in which abuse/neglect had occurred. The *number of subtypes of abuse/neglect* was defined as the total amount of abuse/neglect types (i.e., physical abuse, sexual abuse, neglect) that had occurred from birth to 17.5 years. The subtype *of abuse/neglect* was coded as a binary variable of whether each type of abuse/neglect (i.e., physical abuse, sexual abuse, neglect) had occurred from birth to 17.5 years. Finally, *perpetrator of abuse* was coded as a binary variable of whether physical and/or sexual abuse was perpetrated at any time from birth to 17.5 years by the mother-figure (mother, stepmother, aunt, grandmother), father-figure (father, stepfather, uncle, and grandfather), or non-caregiver. The AAI was administered at ages 19 and 26 years and the CRI was measured at both 20–21 years of age and 26–28 years of age (if participants had multiple CRIs the later CRI was used). Following a set of exploratory factor analyses, attachment representations were operationalized using two dimensions for both the AAI and CRI, respectively: dismissing states of mind and preoccupied states of mind.

A series of hierarchal regressions revealed that overall experiences of abuse/neglect were significantly and uniquely associated with higher ratings of preoccupation during the AAI above and beyond demographic covariates (i.e., child biological sex, child race/ethnicity, family, and maternal education; *β* = .24). AAI preoccupation was uniquely predicted by multiple aspects of abuse/neglect above and beyond demographic covariates and AAI-dismissing states of mind. Specifically the following parameters of abuse/neglect were associated with AAI-preoccupied states of mind: chronicity of abuse/neglect (*β* = .25) and number of abuse/neglect subtypes (*β* = .24), physical abuse (but not sexual abuse or neglect; *β* = .24); father-perpetrated abuse (*β* = .25) and finally, mother-perpetrated abuse (*β* = .22). In contrast, abuse/neglect experiences were not significantly associated with AAI-dismissing states of mind (results held when covariates were included).

When predicting attachment representations regarding one’s romantic partner, neither overall abuse/neglect nor the specific parameters were associated with CRI-preoccupied states of mind. When examining the specific parameters of abuse/neglect only chronicity (*β* = .24) and the number of types of abuse/neglect (*β* = .20) were uniquely associated with CRI-dismissing states of mind above and beyond covariates. Taken together, these results suggested that experiences of child abuse and/or neglect resulted in greater preoccupied states of mind during the AAI and higher dismissing states of mind during the CRI.

Building on Raby et al. (2017), Nivison et al. (2021) examined the predictive significance of abuse/neglect in relation to AAIs that were newly recoded to assess secure base script knowledge (AAI_sbs_; Waters & Facompré, 2021). AAI_sbs_ reflects the extent to which individuals recall their childhood relationships with their primary caregivers to reflect sensitive and responsive attachment figures. To examine the predictive significance of child abuse/neglect beyond a simple binary variable of whether abuse had occurred, Nivison and colleagues used an overall *severity score* originally calculated by Labella et al. (2019). This score is a quasi-continuous scale calculated by summing the number of types of abuse (physical abuse, sexual abuse, neglect) during each developmental period (infancy, early childhood, middle childhood, adolescence). Because each subtype was coded dichotomously for each developmental period, the total experiences of abuse and/or neglect measure had a theoretical minimum of zero (i.e., the participant did not experience any type of abuse or neglect from infancy to adolescence) and a theoretical maximum of 12 (i.e., a participant experienced all three subtypes during each developmental period from infancy to adolescence). Additionally, specific parameterizations of abuse (i.e., perpetrator, developmental timing, subtype) were examined. The perpetrator and subtype variables were consistent with Raby et al. (2017) in that developmental timing was operationalized as whether abuse/neglect had occurred during each developmental period. AAI_sbs_ scores were then aggregated across 19 and 26 years of age to create a composite measure of secure base script knowledge in young adulthood. The demographic covariates were also consistent with Raby et al. (2017).

A series of hierarchal linear regressions revealed that higher rates of child abuse/neglect were associated with lower AAI_sbs_ in young adulthood, above and beyond the demographic covariates and observer-rated maternal sensitivity (*β* = −.18). Overall abuse/neglect, abuse perpetrated by mothers (*β* = −.18), abuse/neglect occurring in middle childhood (*β* = −.20) and adolescence (*β* = −.17), and physical abuse (*β* = −.29) were all uniquely associated with lower secure base script knowledge in young adulthood when controlling for demographic covariates and maternal sensitivity.

To expand the work of Raby et al. (2017) and Nivison et al. (2021), another study was conducted to investigate whether childhood experiences of abuse/neglect predicted secure base script knowledge in romantic relationships using recoded CRIs (CRI_sbs_; DeWitt et al., 2022; Nivison et al., 2022). The abuse/neglect variables were identical to those used by Nivison et al. (2021). However, neither total experiences of abuse/neglect nor any of the specific parameterizations of abuse significantly predicted later CRI_sbs_ when controlling for maternal sensitivity and the demographic covariates.

### Child abuse/neglect and adult psychopathology

VanMeter et al. (2021) investigated the extent to which childhood experiences of abuse/neglect were associated with symptoms of psychopathology in young adulthood. The same prospective abuse/neglect variables from Nivison et al. (2021) were examined. Symptoms of psychopathology were assessed via the Achenbach System of Empirically Based Assessment (ASEBA; Achenbach et al., 2003) at ages 23, 26, 32, and 39 years of age, which were then composited to create a measure of psychopathology in young adulthood. Analyses focused on the total psychopathology measure. Covariates included the standard set of demographic variables as well as total symptoms of psychopathology measured at 16 years of age. A series of linear regressions were conducted including the abuse/neglect variables, psychopathology at age 16, and the demographic covariates.

Overall severity of abuse/neglect was significantly associated with higher total symptoms of psychopathology (*β* = .25). When examining specific parameters, only abuse perpetrator by mothers (*β* = .17) was associated with greater total symptoms of psychopathology. In sum, more experiences of abuse/neglect in childhood, particularly abuse perpetrated by mothers, were associated with more symptoms of psychopathology in young adulthood, above and beyond the demographic covariates and symptoms of psychopathology at age 16 years.

Using the same subsample as Raby et al. (2017), Martin et al. (2017) investigated whether adult attachment as measured by both the AAI and CRI mediated the link between experiences of child abuse/neglect and non-suicidal self-injury (NSSI). Although the CRI subsample was consistent with Raby et al. (2017), the AAI subsample differed in that Martin et al. (2017) focused on the age 19-year AAIs for the primary analysis (but conducted sensitivity analyses with the 26-year AAI). Child abuse/neglect was operationalized as a binary variable of whether any kind of abuse/neglect had occurred, regardless of perpetrator, between birth and 17.5 years of age. The same four demographic covariates consistently used in the MLSRA were included. Childhood experience of abuse/neglect was significantly associated with NSSI by age 26 (*r* = .25), replicating earlier work reported by Yates et al. (2008). Furthermore, overall experiences of abuse/neglect were significantly and uniquely associated with age 19 AAI-preoccupied states of mind (but not AAI-dismissing states of mind), above and beyond covariates (*β* = .29). Adjusting for demographic covariates, AAI-preoccupied states of mind at ages 19 and 26 accounted for significant proportions (21%–29%) of the variance in the associations between child abuse/neglect and NSSI at age 26 years (including demographic covariates). Mediation effects were small in magnitude (κ^2^ = .06 −.07). Although CRI-preoccupied states of mind were significantly associated with child abuse/neglect at the bivariate level (*r* = .24), this association was attenuated to nonsignificant following the inclusion of CRI-dismissing and the covariates. Thus, CRI was not explored as a potential mediator between child abuse/neglect and NSSI. In sum, preoccupied states of mind involving childhood relationships with caregivers partially accounted for the association between child abuse/neglect and NSSI in young adulthood.

### Child abuse/neglect and romantic functioning in adulthood

Labella et al. (2018) investigated potential ties between child abuse/neglect and romantic functioning in adulthood. The abuse/neglect variables were the same as those used by Raby et al. (2017): binary abuse/neglect, chronicity, number of subtypes, perpetrator, developmental timing, and subtype. Romantic functioning was assessed using interviews, observations, and self-reports between 20 and 32 years of age. A principal components analysis yielded two overarching dimensions: romantic competence (defined by interview-rated effectiveness of romantic engagement, observed quality of interactions with romantic partners, and self-reported relationship satisfaction) and relational violence (defined by self-reported perpetration of and/or victimization by relationship violence). The results of a series of hierarchal regressions revealed that experiences of abuse/neglect were significantly associated with both romantic competence (*β* = −.23) and relational violence (*β* = .24) above and beyond the demographic covariates. In both cases, when the opposite relational factor (i.e., relational competence or violence) was entered in the third step, the association between abuse/neglect and relational outcomes the association weakened between abuse/neglect and relational outcomes. The authors concluded that this weakened association was due to a shared association, most likely because of the covariation reflected between romantic competence and relational violence.

Overall, physical abuse (*β* = .20), abuse/neglect chronicity (*β* = −.24), number of types of abuse (*β* = −.22), and abuse perpetrated by mothers (*β* = −.17) were uniquely associated with romantic competence, above and beyond the demographic covariates and relational violence. Only abuse perpetrated by non-caregivers (*β* = .24) was uniquely associated relational violence, above and beyond the covariates and romantic competence. Post hoc analyses revealed that physical abuse by a maternal perpetrator predicted lower romantic competence whereas sexual abuse perpetrated by a non-caregiver predicted higher relational violence. In sum, experiences of child abuse/neglect predict general relational functioning in adulthood, but they more strongly predict relational competence than relational violence.

Zamir et al. (2018) investigated the extent to which symptoms of dissociation in young adulthood mediate the link between child physical and sexual abuse and experiencing intimate partner violence (IPV) in adulthood. The analysis focused on physical and sexual abuse (but not neglect) experienced by female participants (*n* = 80). Abuse was coded as a binary variable of whether physical and/or sexual abuse had ever occurred between birth and 17.5 years of age. Dissociation was assessed via the Dissociative Experiences Scale (Carlson & Putnam, 1993) at age 19. Experiencing IPV was assessed via the Conflict Tactics Scale (Straus, 1979) at ages 23, 26, and 32, which were then aggregated into a composite measure of IPV (perpetrating IPV was not included in this study). The standard set of MLSRA demographic covariates were also included. Child abuse was significantly associated with both dissociation (*r* = .24) and IPV (*r* = .30). Dissociation at age 19 explained 21.9% of the total effect between child abuse and IPV in adulthood. Post hoc analyses that examined physical and sexual abuse separately revealed that the mediation through dissociation was not driven by either type of abuse. Further post hoc analyses revealed that the timing of abuse (i.e., infancy/early childhood vs. middle childhood/adolescence) did not predict either dissociation or IPV in adulthood. In sum, dissociative symptoms may be one mechanism through which individuals who experienced child abuse become at risk for revictimization (i.e., IPV) in adulthood.

### Child abuse/neglect and second-generation parenting

Building on the analysis by Labella et al. (2018), Labella et al. (2019) investigated whether adult relational experiences mediated the association between participants’ own experiences of child abuse/neglect and their parenting quality with their own child. This study focused on the two main relational components from Labella et al. (2018): romantic competence and relational violence. Parenting outcomes were assessed using semi-structured interviews and parental self-reports of CPS involvement when parents were 32 years old, as well as observed parent–child interactions when children were 24- and 48-month of age. Based on prior data reduction efforts (Shlafer et al., 2015), composites were calculated to index interview-rated and observed supportive parenting. The authors of this study created an overall abuse/neglect experiences severity scale consistent with Labella et al. (2019) Finally, the standard set of MLSRA demographic variables was accounted for.

Results indicated that when the participants had greater exposure to childhood abuse/neglect they were less likely to offer supportive parenting to their own children both observed and interview-rated (both *r*s = −.26), as well as have a greater risk of being involved with CPS (*r* = .39). Mediation analyses were conducted for all three parenting outcomes (observed supportive parenting, interview-rated supportive parenting, and CPS involvement); separate analyses were conducted with relational competence and relational violence as the mediators for a total of six mediation analyses. Romantic competence (but not relational violence) partially mediated the association between abuse/neglect exposure and supportive parenting (both observed and interview-rated) above and beyond demographic covariates. Both relational competence and relational violence partially mediated the association between child abuse/neglect exposure and CPS involvement; when both relationship variables were included in a single model, only relational violence emerged as a significant mediator. In sum, these results provide evidence regarding the mechanisms by which experiences of child abuse/neglect may be carried forward to affect parenting in the next generation, via romantic relationship quality. Specifically, child abuse/neglect exposure predicted lower supportive parenting through poorer romantic competence, whereas child abuse/neglect exposure predicted higher CPS involvement through higher rates of violence in romantic relationships in the MLSRA.

### Enduring associations between childhood abuse/neglect and academic and social outcomes

Raby et al. (2019) tested whether experiences of abuse/neglect during infancy and early childhood have transient or enduring effects on social and academic competence across childhood, adolescence, and into adulthood. The transient, or revisionist model, assumes that the effects of early experience on developmental adaptation will get smaller over time, approaching zero in the asymptotic limit. The enduring effects model contrastively assumes that the effects of early experience will be relatively constant over time and will not approach zero in the asymptotic limit (Fraley et al., 2013). To test these two theoretical models, a summary scale of abuse and neglect was calculated for infancy/early childhood as well as later childhood and adolescence by summing the number of types of abuse/neglect that had occurred during those two broad periods. Each scale had a theoretical minimum of zero (no abuse/neglect in either developmental period) and a theoretical maximum of six (all types of abuse and neglect occurred in both developmental periods). Sensitivity analyses examined experiences of abuse/neglect with a binary variable of whether abuse/neglect had occurred. In childhood, teachers rated peer competence at kindergarten, Grades 1, 2, 3, and 6, and at age 16 years. In adulthood, social competence was assessed via interviews on the effectiveness of engagement in romantic relationships at ages 23 and 32 years. In childhood, academic competence was measured via the Peabody Individual Achievement test in Grades 1, 2, 3, and 6 and the Woodcock Johnson Tests of Achievement at age 16 years. In adulthood, academic competence was measured via participants’ self-reported educational attainment at ages 23, 26, 28, 32, and 34 years. Consistent with standard practice in the MLSRA, a set of four demographic covariates was included as well as an aggregate measure of maternal sensitivity assessed at 3, 6, 24, and 42 months of age.

Bivariate associations revealed consistent significant associations between early abuse/neglect experiences and social and academic competence from childhood through adulthood (average *r*s were −.26 and −.32 for social and academic competence, respectively). The results of the modeling approach first described by Fraley et al. (2013) revealed that the enduring model fit the data better than the revisionist model when predicting both social competence (Δ*χ*^2^ = 31.53, *p* < .01) and academic competence (Δ*χ*^2^ = 10.11, *p* < .01). In other words, early abuse and neglect had long-term and enduring associations with less effective social competence and lower educational attainment in adulthood. The average estimate of the direct path from early abuse/neglect was *β* = −.16 (p < .01) for social competence and *β* = −.04 (p < .01) for academic competence. These findings were consistent when controlling for later experiences of child abuse/neglect. Moreover, the enduring association between early abuse/neglect and *social competence* remained significant when controlling for demographic covariates and maternal sensitivity. However, when controlling for demographic covariates and maternal sensitivity, the enduring associations between early abuse/neglect and *academic outcomes* were no longer statistically significant. In sum, early experiences of abuse/neglect have a robust and enduring association with interpersonal relationships across the life span. In contrast, abuse/neglect experiences no longer demonstrated an enduring association with academic competence when maternal sensitivity was included in the model.

### Child abuse/neglect and physical health

Johnson et al. (2017) examined potential ties between child abuse/neglect and adult physical health at 37–39 years of age. Four indicators of physical health were examined and included physical health biomarkers, self-reported physical health ratings, and self-reported health problems. Objective health was assessed by a measure of cardiometabolic risk (based on inflammatory markers that included c-reactive protein (CRP), body mass index, waist-to-hip ratio, and blood pressure). All physical health markers were assessed at age 37 years and only CRP was assessed at age 39 years. Self-reported health was assessed by participants’ ratings of the quality of their overall physical health at age 37 years by asking participants to rate on a 5-point Likert scale, “How would you rate your overall physical health?” Participants also listed health problems (e.g., heart attack, diabetes, cancer) they experienced at age 37 years. Participants’ overall health problems were summed to create a total number of health problems at age 37 years. Child abuse/neglect was operationalized as a binary variable of whether abuse/neglect had occurred anytime from birth to 17.5 years and by examining specific subtype (i.e., physical abuse, sexual abuse, physical neglect). Covariates included the standard MLSRA demographic covariates their self-reported eating, sleeping, and exercise behaviors at age 32 years. Participants with higher overall abuse/neglect histories were significantly more likely to have greater cardiometabolic risk (*r* = .20), self-report having lower quality physical health (*r* = −.27), and have a greater number of health problems (*r* = .19) in adulthood.

Hierarchical linear regressions were conducted to examine the predictive significance of the subtypes of abuse/neglect (entered simultaneously) on each adult health marker. The first step included all three subtypes of abuse/neglect, the second step included the demographic covariates, and the final step included self-reported health behaviors at the age of 32 years. Neglect was uniquely associated with greater cardiometabolic risk (*β* = .25), lower quality self-reported health ratings (*β* = −.27), and more reported total health problems (*β* = .33), above and beyond covariates. Neither physical nor sexual abuse uniquely predicted any markers of adult physical health beyond covariates. In sum, experiencing any kind of abuse and/or neglect during childhood was associated with later physical health problems in adulthood, but neglect appears to be a stronger risk factor for physical health problems. The unique predictive significance of childhood neglect for adult health problems might be attributable to childhood physical neglect being associated with insufficient medical care and/or lack of access to nutritious foods.

Childhood abuse and neglect were also examined in both the MLRSA and a larger study—the Future of Families and Child Wellbeing Study (formerly called the Fragile Families and Child Wellbeing Study, or FFCSW)—investigating the behavioral, cognitive, and socioemotional pathways from early adversity (0–5 years of age) to BMI in adulthood (Doom et al., 2023). Path analyses were conducted with the FFCWS, but since the MLSRA was underpowered for path analyses, linear regression analyses were used to test whether associations reported in FFCWS replicated in MLSRA. Adversity was investigated with four key indicators: socioeconomic status (SES), childhood unpredictability, childhood threat (including abuse), and childhood deprivation (including neglect). SES was measured via socioeconomic index at the prenatal assessment and when children were in early childhood (children aged 42 and 54 months old) and aggregated into a composite measure of SES. Unpredictability was measured via maternal report and included changes in residence, cohabitation, and employment assessed when their child was 12, 18, 48, 54, and 64 months old, aggregated into a measure of unpredictability. The *threat scale* was the sum of binary indicators of whether physical and/or sexual abuse had occurred during infancy or early childhood (possible range: 0–4). *Deprivation* was the sum of binary indicators of whether neglect had occurred during infancy or early childhood (possible range: 0–2). BMI was assessed via self-reports at age 32 years and directly measured in the lab at age 37 years. Additionally, the authors also examined associations between child abuse/neglect and impulsivity, emotion dysregulation, and overeating as potential pathway variables. Impulsivity, emotion dysregulation, and overeating were all measured by the Child Behavior Checklist (CBCL). Impulsivity and emotion dysregulation were assessed at age 5 years and overeating was assessed at age 16 years. Children’s biological sex and ethnicity were included as covariates.

Experiences of threat (i.e., abuse in infancy and early childhood) were significantly associated with both impulsivity (*r* = .26) and emotion dysregulation (*r* = .26) at age 5 years at the bivariate level, but not overeating at age 16 or BMI at ages 32 or 37 years. Similarly, early deprivation (i.e., neglect in infancy and early childhood) was significantly associated with impulsivity (*r* = .22) and emotion dysregulation (*r* = .22) at age 5 years at the bivariate level, but not overeating at age 16 or BMI at age 32 or 37 years. A series of linear regressions were also conducted wherein all four markers of adversity as well as child sex and ethnicity were entered simultaneously as predictors of (1) emotional dysregulation at age 5, (2) impulsivity at age 5, (3) overeating at age 16, (4) BMI at age 32, and (5) BMI at age 37 years. The occurrence of abuse within the first five years of life was uniquely associated with emotion dysregulation (*β* = .20) and impulsivity at age 5 (*β* = .18). No other unique associations between abuse and overeating or BMI emerged. Moreover, there were no associations between neglect and any of the outcome variables. However, greater emotion dysregulation at age 5 predicted more overeating at age 16 and more which in turn predicted higher BMI at ages 32 and 37 years; unexpectedly, more emotion dysregulation at age 5 also predicted lower BMI at age 37 years. In sum, the authors concluded that these results, in combination with the findings from the FFCWS, reveal that impulsivity, emotion dysregulation, and eating behaviors may mediate the link between child abuse/neglect and BMI in adulthood.

## What we have learned about maltreatment from the MLSRA and clinical implications

Tables 2 and 3 contain summary information regarding the associations between abuse/neglect (both omnibus [Table 2] and individual parameter variables [Table 3]) and the outcomes assessed in the studies discussed above. Additional studies have focused on the mechanisms by which abuse/neglect may be carried forward across the lifespan. For example, among individuals with histories of childhood abuse/neglect, AAI preoccupation is associated with NSSI; dissociation is as a risk factor for IPV; lower romantic competence is a risk factor for low quality supportive parenting and CPS involvement; and romantic violence is a risk factor for CPS involvement. Preliminary evidence also suggests that individuals with histories of child abuse/neglect are more likely to be impulsive during childhood and have higher rates of emotion dysregulation, which in turn predicts overeating in adolescence and higher BMI in adulthood. These studies showcase the importance of investigating the specific pathways and/or mechanisms by which abuse/neglect experiences has a negative, cascading effect on well-being across the life span.

The MLSRA has likewise provided extensive evidence elucidating long-term outcomes associated with early experiences of child abuse and neglect. In particular, research has demonstrated the effects of childhood abuse/neglect on multiple aspects of functioning including mental and physical health, interpersonal relationships including parenting, the effects of specific types of abuse/neglect on development, and emerging evidence of mechanisms by which abuse/neglect may be carried forward across the life span. This nuanced investigation of sequalae associated with child abuse/neglect provides insight regarding potential prevention and intervention targets and strategies. For example, MLSRA research demonstrates links between histories of neglect and higher rates of negative physical health issues in adulthood, suggesting that intervening early to prevent and mitigate neglect (e.g., by ensuring hygienic living conditions, providing medical care to under-served families, and promoting children’s access to adequate nutrition) may have downstream implications for reducing disease burden in adulthood. Longitudinal research demonstrating links between abuse/neglect and downstream psychopathology (e.g., internalizing and externalizing symptoms, dissociation, NSSI) indicates an urgent need for clinical assessment and appropriate intervention (e.g., trauma-focused cognitive behavioral therapy) for children identified as experiencing abuse and neglect. Nuanced analyses may guide intervention by elucidating aspects of abuse/neglect experiences that confer specific risk: for example, abuse perpetrated by a maternal caregiver during infancy may suggest the need for long-term follow-up to mitigate risk of externalizing symptoms in adulthood (VanMeter et al., 2021). In addition to identifying populations at risk, the MLSRA may help to inform targets of intervention: abuse and neglect experiences have been found to predict early emotion dysregulation (e.g., Doom et al., 2023; Egeland et al., 1988) and problems in social relationships (e.g., Raby et al., 2019), suggesting that these may be productive targets for early intervention.

Results may also inform efforts to interrupt intergenerational patterns of maltreatment and enhance parenting in the next generation. Consistent with evidence that MLRSA mothers with emotionally satisfying adult relationships were more likely to ‘break the cycle’ of maltreatment (Egeland et al., 1988), analyses with MLSRA offspring identified romantic relationship functioning as a key predictor of parenting outcomes (Labella et al., 2019). Whereas romantic competence uniquely mediated links between abuse/neglect experiences and lower supportive parenting, involvement in relationship violence mediated associations with self-reported CPS involvement. Taken together, this suggests that parents with childhood histories of abuse and neglect may benefit from preventive interventions that (a) enhance supportive romantic and co-parenting relationships, and (b) provide psychoeducation, resources, and conflict resolution strategies for individuals experiencing (or at risk for) relational violence. Overall, MLRSA findings inform cross-disciplinary efforts to promote the well-being and improve the physical and mental health of individuals with adverse early experiences.

## Future directions of the MLSRA

Data collection for the MLSRA is still ongoing as the participants are in their mid to late 40s. Therefore, we anticipate that we will be able to continue to investigate the legacy of early abuse/neglect experiences well into middle adulthood. Our current data collection is focused on assessing the neurobiological, cognitive, and epigenetic indicators of aging (led by Drs. Elizabeth Carlson and Kathleen Thomas). Given our extensive prospective longitudinal data we are eager to investigate how experiences of child abuse/neglect may impact adult functioning and aging trajectories. We will also be able to examine how the ways in which experiences of childhood abuse/neglect are measured (i.e., retrospective vs. prospective) may influence our understanding of the sequelae of maltreatment experiences as there are newly recoded interview-based retrospective reports of childhood maltreatment (Nivison, 2023) in addition to retrospective self-report measures of childhood experiences of maltreatment in the new wave of data collection in the MLSRA. Taken together, we will be able to examine ways in which we can use the “new” retrospective abuse data in combination with the neurobiological data we are collecting to study the midlife legacy of early abuse in the domain of accelerated biological aging.

Beyond these contributions to research on childhood abuse and neglect, the MLSRA continues to advance the developmental psychopathology framework through its commitment to key principles articulated by Dr Dante Cicchetti (Cicchetti & Cohen, 1995; Cicchetti, 2006). From its inception, the MLSRA has examined both normative and atypical development and assessed constructs at multiple levels of analysis, from biological to behavioral to interpersonal constructs. Future research with the MLSRA sample is uniquely situated, not only to examine potential long-term effects of childhood abuse and neglect, but to elucidate trajectories of adult development in multiple domains that are salient during early and middle adulthood. To date, we have leveraged longitudinal data from four decades to ask a myriad of questions from a developmental psychopathological framework, including how life stress from different developmental periods predicts cortisol reactivity in adulthood (Young et al., 2021) and how early attachment security is associated with social and academic outcomes through midlife (Nivison et al., 2023). Furthermore, when the current data collection is complete, we will be able to examine biological, psychological, social, and contextual predictors of trajectories of aging. The MLSRA continues to push the field of developmental psychopathology forward, asking critical questions about typical and atypical development, using longitudinal data collected prospectively from multiple informants, with multiple methodologies, incorporating multiple levels of analysis.

## Conclusion

The MLSRA has contributed in significant ways to the literature on child maltreatment over the last 40 years. The MLSRA was one of the first prospective longitudinal studies of development in the context of risk, initially challenging many of the early assumptions regarding child maltreatment (e.g., maltreatment evoked by child characteristics) and subsequently documenting the impact of early abuse and neglect across development. The MLSRA has contribution to understanding the predictors of childhood maltreatment and its ramifications on various aspects of human development, including cognitive, personality, behavioral, interpersonal functioning, and mental and physical health. It elucidated the intergenerational consequences of childhood maltreatment and revealed underlying mechanisms and protective factors against its detrimental effects on human development. The MLSRA project was initiated by and has been conducted under the leadership of Byron Egeland (with colleagues Alan Sroufe, Andrew Collins, Elizabeth Carlson, Michelle Englund, Jeff Simpson, and Glenn Roisman), and it has also been guided by the work and consultation of Dante Cicchetti. Grounded squarely within a developmental psychopathology perspective, Dr Cicchetti’s seminal maltreatment research (e.g., Barnett et al., 1993; Cicchetti, 2004, 2016) has inspired and informed MLSRA investigations of the impact of childhood adversity adverse caregiving on well-being across the life span.

## Figures and Tables

**Table 1.** Summary of abuse/neglect papers since the recoding of the MLSRA prospective abuse/neglect data

Study	Title	Sample Size	Abuse/neglect variables	Outcome variables examined
DeWitt et al. (2022)	Childhood abuse, neglect, and maternal sensitivity as antecedents of scripted romantic attachment representations	116	Summary abuse/neglect scale, Perpetrator, Developmental timing, Subtype	Secure base script knowledge in the Current Relationships Interview (CRI) at ages 20–21; 26–28 years
Doom et al. (2023)	Behavioral, cognitive, and socioemotional pathways from early childhood adversity to BMI: Evidence from two prospective, longitudinal studies	267	Overall physical abuse & Sexual abuse scale in first 5 years of life	BMI (32 and 37 years), emotion dysregulation, impulsivity at age 5, and overeating at age 16 years
			Overall neglect Scale in first 5 years of life	
Johnson et al. (2017)	Childhood abuse and neglect and physical health at midlife: Prospective, longitudinal evidence	115	Subtype	Cardiometabolic risk, self-reported health, and number of health problems at age 37 years
Labella et al. (2018)	Multiple dimensions of childhood abuse and neglect prospectively predict poorer adult romantic functioning	179	Binary ever abused/neglected, number of subtypes, chronicity, perpetrator, subtype	Component measure of romantic competence and romantic violence assessed from ages 20 to 32 years
Labella et al. (2019)	Romantic functioning mediates prospective associations between childhood abuse and neglect and parenting outcomes in adulthood	122	Summary abuse/neglect scale	Observed supportive parenting (when child was 24- and 42 months), interview-rated supportive parenting and self-reported CPS involvement at age 32
				Mediators: component measure of romantic competence and romantic violence assessed from ages 20 to 32 years,
Martin et al. (2017)	Childhood abuse and neglect, attachment states of mind, and non-suicidal self-injury	164	Binary ever abused/neglected	Non-suicidal self-injury up to 26 years of age
				Mediator: Adult Attachment Interview (AAI) at age 19 years, CRI at 20–21 years; 26–28 years
Nivison et al. (2021)	Childhood abuse and neglect are prospectively associated with scripted attachment representations in young adulthood	157	Summary abuse/neglect scale, Perpetrator, Developmental timing, Subtype	Secure base script knowledge in the AAI at 19 and 26 years of age
Raby et al. (2017)	Childhood abuse and neglect and insecure attachment states of mind in adulthood: prospective, longitudinal evidence from a high-risk sample	164	Binary ever abused/neglected, number of subtypes, chronicity, perpetrator, subtype	Attachment states of mind in adulthood measured via the AAI at 19 and 26 years of age
Raby et al. (2019)		267	Summary abuse/neglect in infancy and early childhood as well as abuse/neglect in middle childhood and adolescence	Social competence (teacher rated) at Kindergarten Grades 1, 2, 3, 6 romantic engagement at 23 and 26 years of age
				Academic competence measured via the PIAT at grades 1, 2, 3, 6 and via the WJR-r at age 16 years
VanMeter et al (2021)	Childhood abuse and neglect and self-reported symptoms of psychopathology through midlife	140	Summary abuse/neglect scale, perpetrator, developmental timing, subtype	Total symptoms of psychopathology, internalizing and externalizing behaviors assessed via Achenbach self-reports at ages 23, 26, 32, and 39 years.
Zamir et al (2018)	The role of dissociation in revictimization across the life span: A 32-year prospective study	80**	Binary ever abused*	Intimate partner violence assessed at ages 23, 26, 32
				Mediator: symptoms of dissociation at age 19 years

*Note*: For clarity, only the target analytic variables are presented in the table. Some studies report more variables than included here. These variables are not reported in the table if they were not related to abuse/neglect analyses or were used in sensitivity analyses.

* Binary ever abused variable is a measure of whether physical or sexual abuse ever occurred and does not include neglect.

** Zamir et al. (2018) included only female participants.

**Table 2.** Summary of all MLSRA overall child abuse/neglect findings

Abuse/Neglect Variables	Outcome variables	
	
Omnibus variables	Child outcome	Adult outcome
Overall abuse/neglect	• Lower peer social competence	• Lower secure base script knowledge derived from the adult attachment interview

		• Lower romantic relational competence

		• Lower quality physical health in adulthood

		• Lower supportive parenting

		• Higher preoccupied attachment states of mind

		• Higher symptoms of internalizing and externalizing behaviors

		• Higher rates of non-suicidal self-injury

		• Higher levels of dissociation

		• Higher levels of intimate partner violence

		• Higher odds of involvement with CPS

**Table 3.** Summary of all specific parameter of the child abuse/neglect findings

Parameters of abuse/neglect	Child outcome	Adult outcome
Chronicity of abuse/neglect		• Higher preoccupied attachment representations
		• Higher dismissing romantic attachment states of mind
		• Lower romantic competence
Number of subtypes		• Higher preoccupied attachment representations
		• Lower romantic relationship attachment quality
		• Lower romantic competence
Overall physical and sexual abuse	• Higher impulsiveness	
	• Higher emotion dysregulation	
Physical Abuse		• Higher preoccupied attachment representations
		• Lower romantic competence
Neglect	• Higher impulsivity	• Higher cardiometabolic risk
	• Higher emotion dysregulation	• Higher number of reported health problems
		• Lower quality self-reported physical health
Mother-perpetrated abuse		• Higher symptoms of total psychopathology
		• Higher symptoms of externalizing behaviors
		• Higher preoccupied attachment representations
		• Lower adult attachment quality (parent–child)
		• Lower romantic competence
Father-perpetrated abuse		• Higher preoccupied attachment representations
		• Lower adult attachment quality (parent–child)
Non-caregiver perpetrated abuse		• Higher symptoms of externalizing behaviors
		• Higher rates of relational violence
Abuse/neglect in infancy/early childhood	• Lower peer social competence	• Lower romantic competence
	• Lower academic achievement	
Abuse/neglect in middle childhood		• Lower adult attachment quality (parent–child)
Abuse/neglect in adolescence		• Lower adult attachment quality (parent–child)
Abuse/neglect in middle childhood and adolescence	• Lower peer social competence	• Lower romantic competence

*Note*: For the parameters of abuse/neglect, the outcome variables presented focused on unique associations. There are no unique associations to report for sexual abuse, abuse/neglect in infancy, or abuse/neglect in early childhood.
